# Clinical significance of interleukin-6, tumor necrosis factor-α and high-sensitivity C-reactive protein in neonates with hypoxic-ischemic encephalopathy

**DOI:** 10.3892/etm.2014.1869

**Published:** 2014-07-29

**Authors:** YUN SHANG, LINA MU, XIXIA GUO, YUHUA LI, LIMIN WANG, WEIHONG YANG, SHUJUN LI, QIONG SHEN

**Affiliations:** 1 Department of Neonatology, The First Affiliated Hospital of Xinxiang Medical University, Weihui, Henan 453100, P.R. China; 2 Department of Pediatrics, The First Affiliated Hospital of Xinxiang Medical University, Weihui, Henan 453100, P.R. China; 3 Department of Pediatric Rehabilitation, The First Affiliated Hospital of Xinxiang Medical University, Weihui, Henan 453100, P.R. China; 4 Department of Pediatric Intensive Care Unit, The First Affiliated Hospital of Xinxiang Medical University, Weihui, Henan 453100, P.R. China; 5 Department of Gynecology and Obstetrics, Hebei Armed Police Corps Hospital, Shijiazhuang, Hebei 050081, P.R. China

**Keywords:** hypoxic-ischemic encephalopathy, interleukin-6, tumor necrosis factor-α, high-sensitivity C-reactive protein, prognosis

## Abstract

The present study aimed to investigate the potential roles of interleukin-6 (IL-6), tumor necrosis factor-α (TNF-α) and high-sensitivity C-reactive protein (Hs-CRP) in the progression and prognosis of neonatal hypoxic-ischemic encephalopathy (HIE). The observation group comprised 74 neonates with HIE and the control group comprised 74 healthy neonates. The serum levels of IL-6, TNF-α and Hs-CRP were measured in the patients with HIE and the normal control infants. The correlations between the variances in the levels of these inflammatory cytokines and the different clinical gradings and prognoses of the disease were analyzed. The data revealed significant upregulation of the serum levels of IL-6, TNF-α and Hs-CRP in patients with HIE. The increase in the levels of these inflammatory mediators correlated with the severity of the disease and also had a positive correlation with the prognosis of the disease. In conclusion, high levels of IL-6, TNF-α and Hs-CRP were observed in neonatal patients with HIE. Thus, these inflammatory mediators may play a role in the progression and prognosis of the disease.

## Introduction

Neonatal hypoxic-ischemic encephalopathy (HIE) causes high infant mortality and long-term morbidity rates (1–3). HIE occurs in ~1–3 per 1,000 full-term infants and in almost 60% of premature newborns. Approximately 15–20% of affected newborns succumb within the postnatal period (4) and an additional 25% develop severe and permanent neuropsychological handicaps (5), including cerebral palsy, seizures, visual impairment, mental retardation, learning disabilities and epilepsy. Following the infiltration of circulating monocytes, neutrophils and T-cells in neonates, cerebral ischemia initiates an immediate innate immune response that may occur minutes following insult. This in turn exacerbates damage to the brain with a large accumulation of inflammatory cytokines (6–8). However, interleukin-6 (IL-6) and tumor necrosis factor-α (TNF-α), two inflammatory cytokines that are accumulated during cerebral ischemia, play a protective role in the incidence of HIE (9,10). Secreted by T cells, B cells and macrophages, IL-6 and TNF-α may be enriched during the progression of stress (11,12). High-sensitivity C-reactive protein (Hs-CRP), an acute-phase protein, is secreted by the liver in response to factors released by macrophages and fat cells. It is a sensitive marker of inflammatory reactions, since its levels rise in response to inflammation (13,14).

To date, several studies have revealed the association of inflammatory cytokines with the process of HIE (11–13); however, it remains unclear as to whether these cytokines play a role in the progression and prognosis of the disease. The current study analyzed the levels of IL-6, TNF-α and Hs-CRP among the different clinical gradings of HIE and further investigated the correlation between the changes in the levels of these inflammatory cytokines and the clinical prognosis of the disease.

## Subjects and methods

### Patients

A total of 74 patients with HIE, admitted to The First Affiliated Hospital of Xinxiang Medical University (Weihui, China) hospital between June 2010 and June 2013, were involved in the current study. All patients had been previously diagnosed and clinically graded based on encephalic computed tomography scans and the clinical determination criteria for HIE (15) (Table I). The control group comprised 74 healthy newborns. There were no statistically significant differences between the HIE and control groups in terms of gender, gestational age and weight (P>0.05). All patients with HIE were classified by clinical grading and comprised 31 individuals with mild, 26 with moderate and 17 with severe HIE. There was no statistically significant difference among the different gradings in terms of gender, gestational age and weight (P>0.05). Furthermore, the patients with HIE were divided into good and poor prognosis groups with 32 and 42 individuals in each group, respectively. Once again, there was no statistically significant difference between the groups in terms of gender, gestational age and weight (P>0.05). The present study was conducted in accordance with the Declaration of Helsinki and with approval from the Ethics Committee of The First Affiliated Hospital of Xinxiang Medical University. Written informed consent was provided by the legal guardians of all participants.

### Treatment methods

All patients were administered narcotic, anti-acidosis, encephalic hypotensive and antioxidant drugs. Hyperbaric oxygen therapy was also performed daily on all patients.

### Enzyme-linked immunosorbent assay (ELISA) and radioimmunometric assay (RIA)

The serum levels of IL-6 and TNF-α were evaluated by ELISA using commercially-available kits (R&D Systems, Minneapolis, MN, USA). A RIA was carried out to detect the serum levels of Hs-CRP using a commercially-available kit (Jokoh Co., Ltd., Tokyo, Japan). The assays were performed following the manufacturers’ instructions.

### Statistical analysis

Computerized statistical analyses were performed using SPSS software version 13.0 (SPSS, Inc., Chicago, IL, USA). Data are presented as mean ± standard deviation. The variances in the levels of the inflammatory cytokines between the controls and patients were analyzed using the Student’s t-test. The comparisons of these parameters among the different grading groups were calculated using analysis of variance (ANOVA) and multiple comparison tests. The correlation between the change in the levels of the inflammatory cytokines and the clinical grading and prognosis of the disease was examined by Spearman’s correlation analysis. In all statistical analyses, a two-tailed P-value ≤0.05 was considered to indicate a statistically significant difference.

## Results

### Serum concentrations of IL-6, TNF-α and Hs-CRP

**Figure 1:**
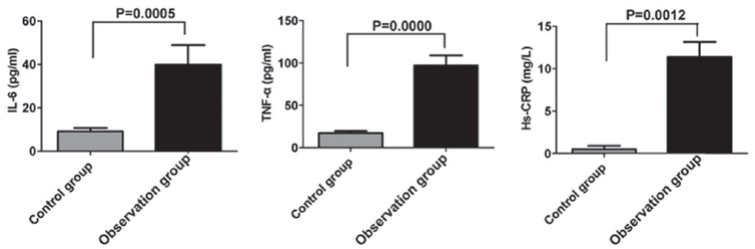
Concentrations of interleukin-6 (IL-6), tumor necrosis factor-α (TNF-α) and high-sensitivity C-reactive protein (Hs-CRP) in the serum. Serum levels of IL-6, TNF-α and Hs-CRP in patients with hypoxic-ischemic encephalopathy (HIE) and normal control infants were evaluated by enzyme-linked immunosorbent assay (Il-6 and TNF-α) or radioimmunometric assay (Hs-CRP).

The serum levels of IL-6, TNF-α and Hs-CRP were detected. The levels of IL-6 (39.94±4.46 pg/ml; P<0.05), TNF-α (97.00±5.97 pg/ml; P<0.05) and Hs-CRP (11.93±1.91 mg/l; P<0.05) were significantly higher in the patients with HIE compared with the respective values in the control group, IL6 (9.18±1.27 pg/ml), TNF α (17.20±1.26 pg/ml) and Hs CRP (0.51±0.18 mg/l) (Fig. 1).

### Comparison of the serum levels of IL-6, TNF-α and Hs-CRP in patients with different clinical gradings

**Figure 2:**
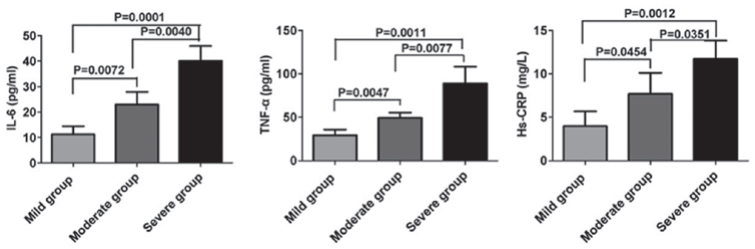
Comparison of the serum levels of interleukin-6 (IL-6), tumor necrosis factor-α (TNF-α) and high-sensitivity C-reactive protein (Hs-CRP) in patients with hypoxic-ischemic encephalopathy (HIE) with different clinical gradings. Serum levels of IL-6, TNF-α and Hs-CRP were analyzed among the groups of patients with mild, moderate or severe HIE.

The present investigation revealed that the upregulation of inflammatory cytokines was accompanied by deterioration of the disease. As shown in Fig. 2, the levels of IL-6, TNF-α and Hs-CRP in the moderate and severe patient groups were significantly higher compared with those in the mild group (P<0.05). Furthermore, there was a significant upregulation of the cytokines in the severe group compared with those in the moderate group (P<0.05).

### Comparison of the serum levels of IL-6, TNF-α and Hs-CRP in patients with different prognoses

**Figure 3:**
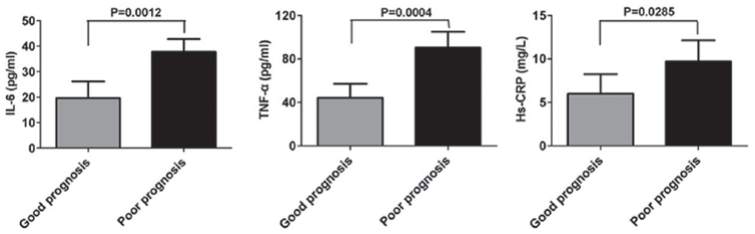
Comparison of the serum levels of interleukin-6 (IL-6), tumor necrosis factor-α (TNF-α) and high-sensitivity C-reactive protein (Hs-CRP) in patients with hypoxic-ischemic encephalopathy (HIE) with different prognoses. Serum levels of IL-6, TNF-α and Hs-CRP were analyzed in patients with a poor or good prognosis.

The levels of IL-6, TNF-α and Hs-CRP in patients with either a poor or good prognosis were further analyzed. Significant upregulation of the levels of IL-6 (37.75±4.24 pg/ml; P<0.05), TNF-α (90.23±7.37 pg/ml; P<0.05) and Hs-CRP (9.71±2.14 mg/l; P<0.05) were observed in patients who had a poor prognosis compared with those in the patients who had a good prognosis, IL 6 (19.59±2.94 pg/ml), TNF α (44.32±4.84 pg/ml) and Hs CRP (5.99±0.99 mg/l) (Fig. 3).

### Correlation between the changes in the levels of inflammatory cytokines and the clinical grading and prognosis of the disease

Based on the variations in the levels of IL-6, TNF-α and Hs-CRP in patients with HIE, it is hypothesized that they may play a role in the progression and prognosis of the disease. A correlation analysis between these changes and the disease progression and prognosis was carried out. Notably, positive correlations were identified between the levels of IL-6, TNF-α and Hs-CRP and the clinical grading (r=1.071, 0.811, 0.704, respectively; P<0.05) and prognosis (r=1.071, 0.811, 0.704, respectively; P<0.05) of the disease.

## Discussion

HIE occurring in fetuses and neonates is a major cause of acute mortality and chronic neurological disability in surviving individuals (16). An increasing number of studies have indicated that there is a complicated correlation between HIE and the immune system (17,18). Cytokines are activated in glial cells and astrocytes in the central nervous system (CNS) and are released in response to brain damage. In return, the activated cytokines regulate the activity of the immune system. Thus, inflammatory cytokines play an important role in brain inflammation caused by the occurrence of HIE. IL-6 is a multifunctional immune mediator that regulates cellular immunity and the inflammatory response. Being a pro- and anti-inflammatory factor, IL-6 has caused controversy in studies investigating its role in HIE in previous years. The ambiguous effects of IL-6 on the CNS have been observed not only in animal models, but also in human studies. The overexpression of IL-6 in animals or its increased release in the brain has neurotoxic effects and may trigger an inflammatory response cascade. By contrast, IL-6 deficient mice demonstrate neuroprotective and anticonvulsive characteristics (19,20). Furthermore, higher levels of IL-6 have been observed in infants with HIE than in normal infants and the concentration of IL-6 has been found to be significantly associated with the severity of HIE and the neurodevelopmental outcome at two years of age (21). Secreted by macrophages, TNF-α is accumulated under stress and manipulates tissue injury. In the CNS, TNF-α is secreted by microglia and astrocytes. Previous studies have indicated that TNF-α may act in a concentration-dependent manner. TNF-α has been reported to play an immunoprotective role in HIE at a low concentration, but exert a proinflammatory effect at a high concentration (22,23). Hs-CRP is an acute-phase protein secreted at a low level by the liver under normal circumstances. It is a sensitive marker of inflammatory reactions since its levels increase in response to inflammation when the body is under stress (24,25).

The present study revealed that the serum levels of IL-6, TNF-α and Hs-CRP in patients with HIE were upregulated when compared with those in the normal controls. The increased levels of Hs-CRP indicate the presence of an acute inflammatory response in the patients; furthermore, the high levels of IL-6 and TNF-α may have evoked an inflammatory response cascade and caused further damage in the brain.

In order to detect the potential correlation between the inflammatory factors and the different clinical gradings of the disease, the serum levels of IL-6, TNF-α and Hs-CRP in patients with different clinical gradings were analyzed. The results demonstrated a correlation between the upregulation of these cytokines and the severity of the disease. Notably, a positive correlation between the grading severity and the different cytokines was identified. Thus, patients with greater inflammatory responses suffered from a severe progression of the disease.

Since a quarter of the patients who survive with HIE suffer from a variety of neuropsychological disabilities, the possible correlation between these mediators and the prognosis of the disease was investigated. Notably, a positive correlation between the different inflammatory factors and the prognosis of the disease was identified.

In conclusion, the current study highlighted the presence of high levels of IL-6, TNF-α and Hs-CRP in patients with HIE and the potential role of these inflammatory mediators in the progression and prognosis of the disease. HIE not only induces the expression of cytokines in the brain but also changes the levels of the peripheral cytokines, which in turn exacerbates the disease in the brain and other tissues.

## Figures and Tables

**Table I:** Clinical information of the patients.

Group	Cases	Gender		Gestational age, weeks (mean±SD)	Weight, g (mean±SD)

		Male	Female		
Control	74	44	30	39.5±2.9	3418±524
HIE	74	42	32	39.6±2.7	3591±619

HIE, hypoxic-ischemic encephalopathy; SD, standard deviation.
